# Temporal Variation in Community Composition of Root Associated Endophytic Fungi and Carbon and Nitrogen Stable Isotope Abundance in Two *Bletilla* Species (Orchidaceae)

**DOI:** 10.3390/plants10010018

**Published:** 2020-12-24

**Authors:** Xinhua Zeng, Haixin Diao, Ziyi Ni, Li Shao, Kai Jiang, Chao Hu, Qingjun Huang, Weichang Huang

**Affiliations:** 1 Shanghai Chenshan Plant Science Research Center, Chinese Academy of Sciences, Chenshan Botanical Garden, Shanghai 201620, China; zengxinhua@csnbgsh.cn (X.Z.); dhxtx1216@163.com (H.D.); niziyi@csnbgsh.cn (Z.N.); shaoli@csnbgsh.cn (L.S.); jiangkai@csnbgsh.cn (K.J.); huchao@csnbgsh.cn (C.H.); 2 Shanghai Institute of Technology, Shanghai 201418, China; huangqingjun@sit.edu.cn; 3 College of Landscape Architecture, Fujian Agriculture and Forestry University, Fuzhou 350002, China

**Keywords:** orchid mycorrhiza, stable isotope, phenological stages, community structure, root-associated endophytes

## Abstract

Mycorrhizae are an important energy source for orchids that may replace or supplement photosynthesis. Most mature orchids rely on mycorrhizae throughout their life cycles. However, little is known about temporal variation in root endophytic fungal diversity and their trophic functions throughout whole growth periods of the orchids. In this study, the community composition of root endophytic fungi and trophic relationships between root endophytic fungi and orchids were investigated in *Bletilla striata* and *B. ochracea* at different phenological stages using stable isotope natural abundance analysis combined with molecular identification analysis. We identified 467 OTUs assigned to root-associated fungal endophytes, which belonged to 25 orders in 10 phyla. Most of these OTUs were assigned to saprotroph (143 OTUs), pathotroph-saprotroph (63 OTUs) and pathotroph-saprotroph-symbiotroph (18 OTUs) using FunGuild database. Among these OTUs, about 54 OTUs could be considered as putative species of orchid mycorrhizal fungi (OMF). For both *Bletilla* species, significant temporal variation was observed in the diversity of root endophytic fungi. The florescence and emergence periods had higher fungal community richness of total species and endemic species than did other periods. Both *Bletilla* species were dominated by Agaricomycetes and Basidiomycota fungi throughout the whole year; however, their abundances varied between two *Bletilla* species and among phenological stages. Meanwhile, the ranges of ^13^C and ^15^N natural abundance were also highly dynamic across all growth stages of *Bletilla* species. Compared with the surrounding autotrophic plants, significant ^13^C enrichments (ε^13^C) were found across all phenological stages, while significant ^15^N enrichment in the florescence period and strong ^15^N depletion during the fruiting period were found for both *Bletilla* species. We can deduce that both *Bletilla* species obtained carbon from root endophytic fungi during the whole year. Additionally, the temporal varying tendency of root endophytic fungal diversity was consistent with ^13^C enrichments, which was also accord with the nutritional requirement of plant.

## 1. Introduction

Orchidaceae is one of the largest plant families, including almost 10% of all angiosperm species and about 28,000 species comprising 736 genera worldwide [1,2]. Members of this family are widely distributed among terrestrial ecosystems, with the notable exception of extremely cold and dry areas and accordingly have a great variety of life history strategies, ranging from epiphytic to terrestrial and from evergreen to achlorophyllous species [3,4]. Zettler et al. reported that unique characteristics of the orchid family, including its high diversity, might be attributable to its distinctive relationships with root endophytic fungi [5]. Although the family is species rich, many orchids are threatened or nearly threatened by extinction, and all wild orchids are listed in the convention on international trade in endangered species of wild fauna and flora [6]. Three main reasons exist for this: A lack of mycorrhizal fungi for seed germination and seedling recruitment [7], a lack of pollinators necessary for sexual reproduction [8], and the impact of human disturbances on many orchid species [9,10].

The tiny dust-like seeds of orchids have room for only marginal amounts of carbon reserves within embryos, and thus, orchids rely on root endophytic fungi (both mycorrhizal and non-mycorrhizal fungi) throughout their life cycle, especially during seed germination and seedling recruitment periods [11,12]. The initial developmental stage of all orchids is a nonphotosynthetic protocorm, totally dependent on C and nutrients supplied by fungal partners [13]. However, all mature orchids, with the exception of a few epiphytic tropical orchids, also maintain mycorrhizal associations throughout the rest of their life cycles [11,14]. Researchers have found that root endophytic fungi can not only transport carbohydrates and break down cellulose in the matrix but can also directly provide nutrients and hormones (e.g., amino acids, gibberellins and jasmonate) for growth [10,15]. In addition, root endophytic fungi were found to promote the absorption of macronutrient elements and micronutrient elements by orchids [16,17] and facilitate the production of metabolites, including antibiotics, phenolic compounds, peroxidase and hydrolase, thus enhancing disease resistance and stress tolerance in orchids [18,19]. Mycorrhizal partners can also influence orchid distributions and determine which habitats allow orchid growth and what environmental factors are critical for orchid recruitment [20].

Interactions with pollinators have shaped orchid floral morphology; similarly, interactions with root endophytic fungi have deeply influenced their trophism [21]. Root endophytic fungal communities in terrestrial ecosystems are diverse and play a vital role in connecting above- and below-ground nutrient cycles [22]. Stable isotope natural abundance analysis, together with molecular identification of mycorrhizal partners, is a powerful approach to assessing the nutritional relationship between mature orchids and their mycorrhizal partners [21,23]. By using stable isotopes, it is possible to evaluate nutrient fluxes under field conditions and trace the source of specific nutrients, through utilizing isotopic differences between plant- and fungus-derived C and N [24,25], enabled by fungal tissues being enriched with ^13^C and ^15^N compared with neighbouring autotrophic plants [26,27]. Meanwhile, different functional groups of fungi forming orchid mycorrhizae can obtain different sources of C and nutrients, thus shaping stable isotope abundance patterns in fungal tissues and their associated orchids [26,28]. Researchers have found that orchids associated with ectomycorrhizal fungi (i.e., achlorophyllous orchid species) [29,30] and saprotrophic wood-decomposers or litter-decaying fungi [31,32] were enriched for ^13^C and ^15^N isotopes in comparison to neighbouring autotrophic plants and that mature partially mycoheterotrophic orchids (i.e., green forest orchids associated with ectomycorrhizal fungi) were positioned between fully mycoheterotrophic orchids and autotrophic plants [23]. In contrast with mycoheterotrophic orchids, stable isotope natural abundance for photosynthetic orchids revealed variation among species [25]. *Listera ovata* and *Orchis purpurea* were found to exhibit ^13^C enrichment [23,33], while significant ^13^C depletion was found in the orchid tribes Orchideae and Cranichideae [34]. However, Gebauer and Meyer found that some rhizoctonia-associated orchids exhibited ^13^C abundance equivalent to that of autotrophic reference plants [23]. 

The trophic relationship between orchids and root endophytic fungi is greatly influenced by many factors, including abiotic factors, such as climate, irradiance, soil nutrients, pH and soil humidity, and biotic factors, such as orchid phylogenetic process, life form and growing period [20,35]. Phenological phase is a key factor regulating mycorrhizal community composition and its trophic relationship with orchids [16,25]. Thus far, trophic relationships between orchids and root endophytic fungi have been investigated in plants collected at single time points, which neglects possible seasonal variation in isotopic signatures that may reflect changes in plant nutritional requirements and variation in nutrient availability throughout the growing season. Meanwhile, mycorrhizal community compositions are often studied in steady-state situations [14,33,36]. It is unclear whether these fungal communities are temporally stable and whether seasonal changes in mycorrhizal associations could also lead to differences in the nutritional status of orchids.

In this study, we used stable isotope natural abundance analysis and DNA molecular identification methods to investigate temporal variation in C and N stable isotope abundance and root endophytic fungi assemblages associated with two mature greenish *Bletilla* spp., *Bletilla striata* (Thunb. ex A. Murray) Rchb. f. and *Bletilla ochracea* Schltr. Our main objectives are to answer the following questions: (1) What are the root endophytic fungi of the two *Bletilla* spp.? (2) Do the composition and abundance of root endophytic fungi and C and N stable isotope abundance of *Bletilla* vary among phenological stages or between the two species?

## 2. Results

### 2.1. Fungal Community Found in Bletilla Roots

**Figure 1:**
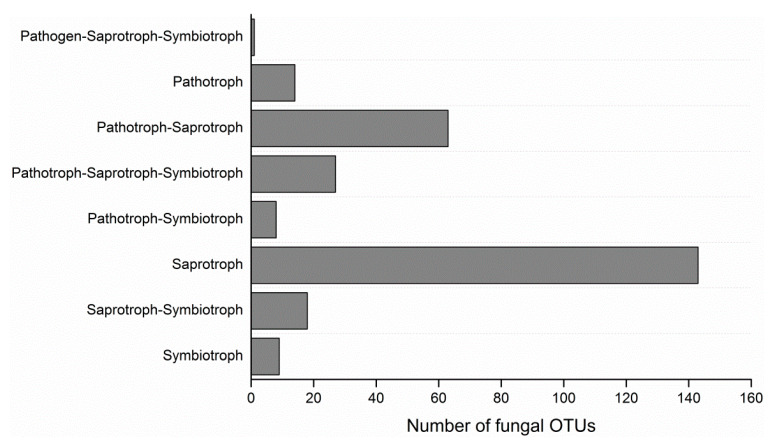
Frequency distribution displaying the number of operational taxonomic units (OTUs) belonging to the different trophic guilds identified in the roots of *Bletilla*.

Illumina MiSeq sequencing yielded a total of 1381,534 sequences with a mean length of 265 bp that passed the quality filtering and could be assigned to the 30 samples. The number of sequences per individual orchid varied from 31,651 to 70,261. A total of 691 operational taxonomic units (OTUs) were identified using a 3% dissimilarity cutoff and removed chimeric sequences as well as global singletons. After discarding non-fungal sequences and data flattened, 467 OTUs could be assigned to root-associated fungal endophytes, which belonged to 25 classes in 10 phyla. The orders with the highest number of OTUs were Hypocreales (41 OTUs), Pleosporales (39 OTUs), Chaetosphaeriales (35 OTUs), Eurotiales (25 OTUs) and Helotiales (24 OTUs). Using FunGuild database, the putative life strategy was assigned only to OTUs with taxonomic assignment at ‘species’ level (283 OTUs). Analyzed OTUs were assigned to saprotroph (143 OTUs), pathotroph-saprotrop (63 OTUs), saprotroph-symbiotroph (18 OTUs), pathotroph-saprotroph-symbiotroph (27 OTUs), pathogen-saprotroph-symbiotroph (1 OTUs), symbiotroph (9 OTUs), pathotrophs (14 OTUs) or pathotroph-symbiotroph guilds (8 OTUs) (Figure 1). Among these OTUs, about 54 OTUs could be considered as putative species of OMF: They were related to Aspergillaceae (*Aspergillus* and *Penicillium*), Saccharomycetales_fam_Incertae_sedis (*Candida*), Nectriaceae (*Fusarium*), Tulasnellaceae (*Tulasnella*, *Epulorhiza*, and one unidentified Tulasnellaceae), Ceratobasidiaceae (*Rhizoctonia*), Glomerellaceae (*Colletotrichum*), Serendipitaceae (*Serendipita*), Pleosporaceae (*Alternaria*), Cucurbitariaceae (*Pyrenochaeta*), Pyronemataceae (*Tricharina*), Omphalotaceae (*Gymnopus*), Hypocreaceae (*Trichoderma*), Myxotrichaceae (*Oidiodendron*), Tricholomataceae (*Mycena*), and Nectriaceae (*Cylindrocarpon*).

### 2.2. Temporal Variation in Diversity of Root Endophytic Fungi

**Figure 2:**
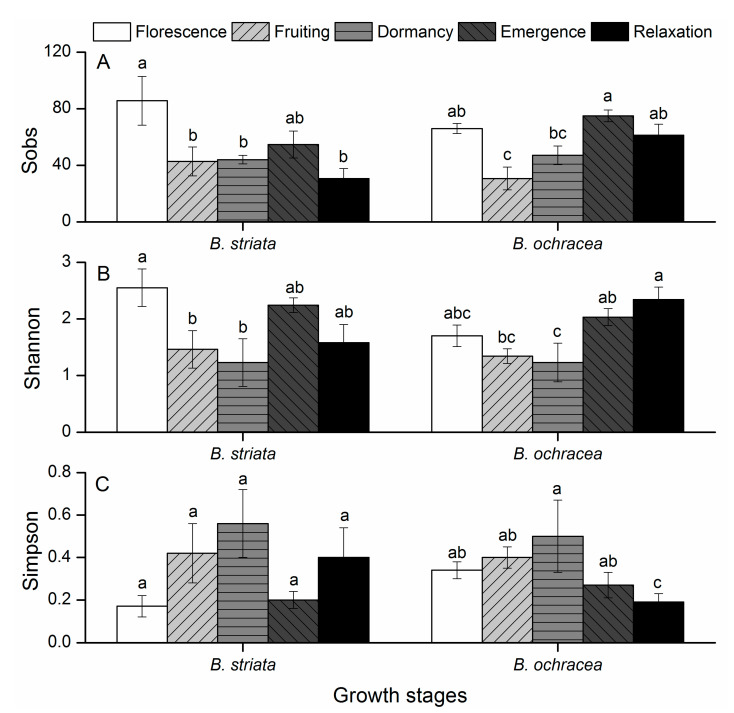
Alpha diversity estimates of mycorrhizal fungi associated with *Bletilla striata* and *B*. *ochracea* at different growth stages. Significant differences (*p* < 0.05) among different growth stages of each species for each index are indicated with lowercase letters. (**A**) Sobs, (**B**) Shannon, (**C**) Simpson.

Fungal community richness (Sobs) and alpha diversity (Shannon and Simpson’s) index values were compared among five phenological stages for the two *Bletilla* species at the operational taxonomic unit (OTU) level (Figure 2). For *B. striata*, the florescence period had significantly higher Sobs and Shannon index values compared with the fruiting and dormancy periods. For *B. ochracea*, the emergency period had significantly higher Sobs value while the relaxation period had significantly higher Shannon index value compared with the fruiting and dormancy periods. In contrast, for both *B. striata* and *B. ochracea*, the dormancy and fruiting periods had higher Simpson’s index values than did the other phenological stages, though the differences were not very significant (Figure 2C). Good’s coverage scores of all phenological stages for the two *Bletilla* species were very high, ranging from 99.95% to 99.99%, indicating that the sequencing depth was adequate to reliably describe the root endophytic fungi associated with *Bletilla* species at different phenological stages, and no significant differences were observed among the five phenological stages for each species (Table S1). 

### 2.3. Community Composition of Root Endophytic Fungi among Different Phenological Stages

**Figure 3:**
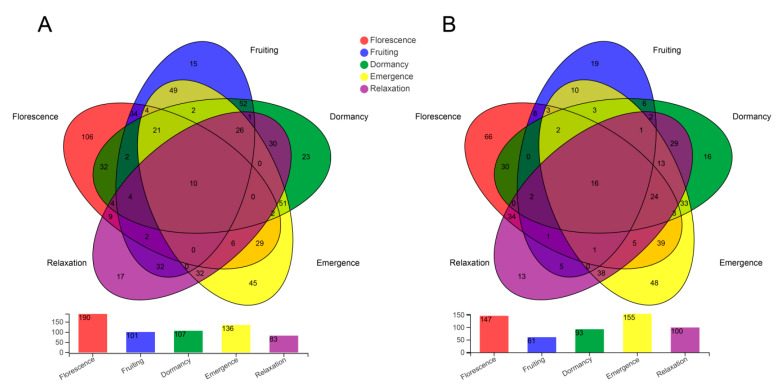
The operational taxonomic unit (OTU) level of species composition during different phenological stages for *Bletilla striata* (**A**) and *B. ochracea* (**B**).

There were 10 and 16 fungal OTUs shared among all five phenological stages in association with *B. striata* and *B. ochracea*, respectively (Figure 3). The shared 10 OTUs for *B. striata* belong to unclassified_c_Agaricomycetes (42.44%), *Exophiala* (19.35%), unclassified_o_Sebacinales (13.05%), *Rhizoctonia* (8.55%), *Neocosmospora* (5.92%), *Dactylonectria* (3.00%), *Cylindrocarpon* (2.75%) and *Aspergillus* (1.97%). The shared 16 OTUs for *B. ochracea* belong to unclassified_c_Agaricomycetes (70.29%), *Exophiala* (11.66%), unclassified_p_Basidiomycota (7.97%), *Paraphoma* (4.12%), *Fusarium* (3.21%), *Neocosmospora* (2.42%), and others (0.34%) (Figure S1). Additionally, there were 106, 15, 23, 45 and 17 fungi OTUs specific to a single stage of florescence, fruiting, dormancy, emergence and relaxation for *B. striata*, respectively, while there were 66, 19, 16, 48 and 13 OTUs, respectively, for *B. ochracea*. There also exist some fungi OTUs shared by 2, 3, and 4 stages for both *B. striata* and *B. ochracea*.

**Figure 4:**
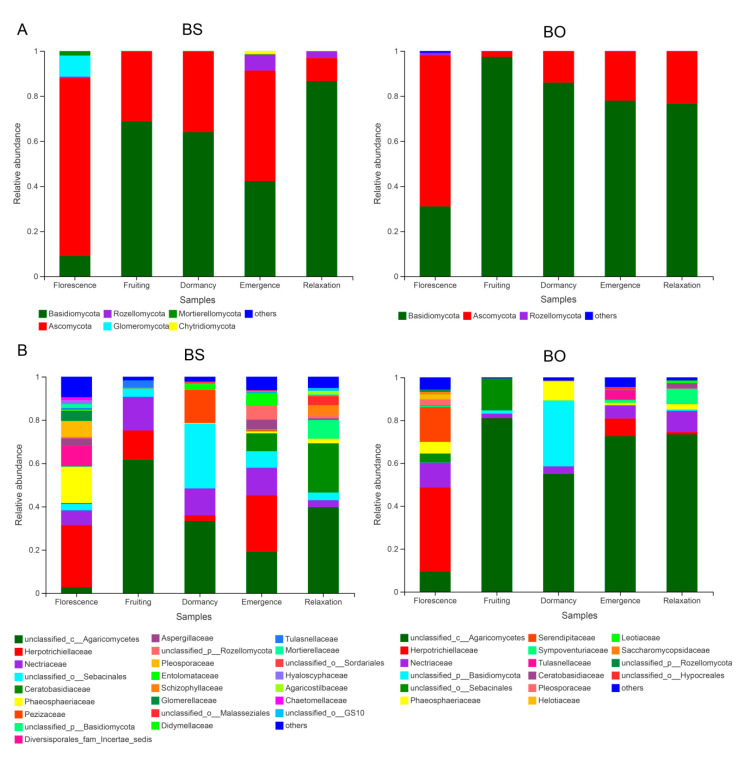
Relative abundances of mycorrhizal fungi in different phenological stages at different phylogenetic levels. (**A**) Relative abundances of mycorrhizal fungi at the phylum level. (**B**) Relative abundances of mycorrhizal fungi at the family level. BS and BO represent *Bletilla striata* and *B. ochracea*, respectively.

The predominant species in root endophytic fungal communities associated with the two *Bletilla* species were largely consistent among the five phenological stages at the phylum level. The fungal phyla with the highest relative abundances were Basidiomycota and Ascomycota. However, differences in relative abundances between the two species for a specific phenological stage or among different phenological stages for each *Bletilla* species were observed. Other minor phyla, such as Rozellomycota, Glomeromycota, Mortierellomycota and Chytridiomycota, were also found for *B. striata*, while only Rozellomycota was found for *B. ochracea* at some phenological stages (Figure 4). At the florescence stage, *B. striata* and *B. ochracea* fungal communities were dominated by Ascomycota, with percentages of 78.93% and 67.07%, respectively, while during the fruiting, dormancy and relaxation stages, *B. striata* and *B. ochracea* fungal communities were dominated by Basidiomycota, with percentages ranging from 63.94% to 86.71% and 76.53% to 97.39%, respectively. However, during emergence, associations with *B. striata* were dominated by Ascomycota (49.04%) and Basidiomycota (42.30%) while associations with *B. ochracea* were domianted by Basidiomycota (77.95%).

At the family level, the florescence stage for both *Bletilla* species had the most diverse associated fungal species, with *B. striata* dominated by Herpotrichiellaceae, Phaeosphaeriaceae. Diversisporales_fam_Incertae_sedis, Pleosporaceae, Nectriaceae and Glomerellaceae, and *B. ochracea* dominanted by Herpotrichiellaceae, Serendipitaceae, Nectriaceae, Phaeosphaeriaceae and unclassfied_o_Sebacinales. Meanwhile, unclassifed_c_Agaricomycetes, Herpotrichiellaceae, Nectriaceae and Phaeosphaeriaceae were present among all five phenological stages for both *Bletilla* species. During the whole physiological period, *B. ochracea* had higher unclassifed_c_Agaricomycetes abundance and lower Herpotrichiellaceae abundance compared with *B. striata*. The florescence stage had higher unclassfied_o_Sebacinales abundance compared with other physiological stages for both *Bletilla* species (Figure 4).

**Figure 5:**
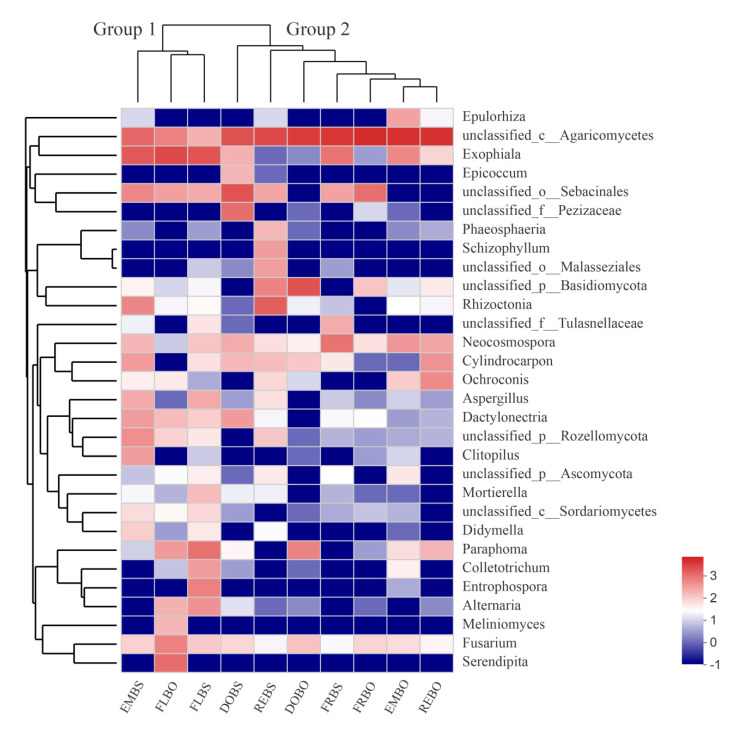
Heatmap of relative abundances of the top 30 fungal genera associated with *Bletilla striata* and *B*. *ochracea* species at different phenological stages. DOBS, *B*. *striata* dormancy; FRBS, *B*. *striata* fruiting; EMBS, *B*. *striata* emergence; REBS, *B*. *striata* relaxation; FLBS, *B*. *striata* florescence; DOBO, *B*. *striata* dormancy; FRBO, *B*. *ochracea* fruiting; EMBO, *B*. *ochracea* emergence; REBO, *B*. *ochracea* relaxation; FLBO, *B*. *ochracea* florescence. Rows are fungal genera, and columns are samples. Colors indicate taxa with a higher (red) or lower (blue) relative abundance in each sample.

At the genus level, as shown by the heatmap in Figure 5, the samples were divided into two groups, and root endophytic fungi communities were divided into four groups. The fungal genera associated with the emergence and florescence stages of *B. striata* and the florescence stage of *B. ochracea* were clustered into one group (group 1), while the other samples were clustered into another group (group 2). Group 1 had higher abundances of *Exophiala*, *Dactylonectria*, unclassified_p_Rozellomycota and *Fusarium* than group 2, while group 2 had higher abundances of unclassified_c_Agaricomycetes than group 1.

### 2.4. Dynamics of Isotopic Abundances over the Bletilla Growth Season

**Figure 6:**
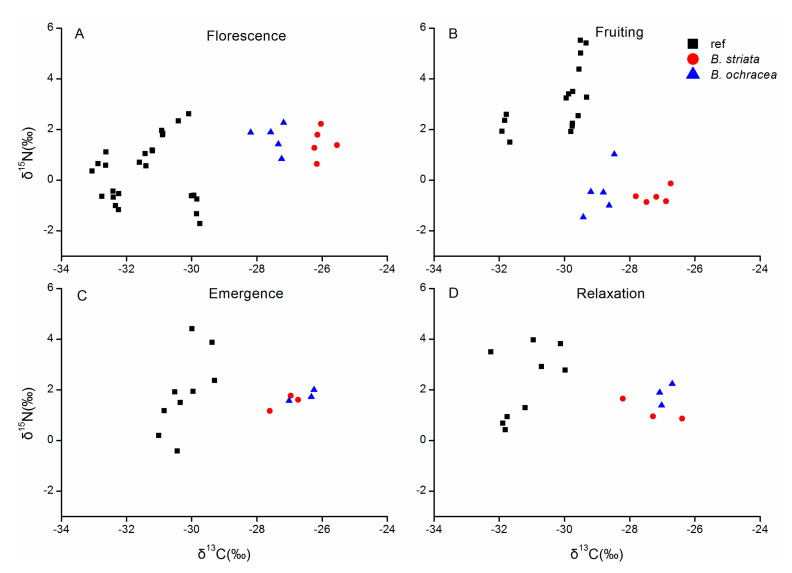
Overview of δ^13^C and δ^15^N values of two *Bletilla* species and autotrophic reference plants (ref) at four different phenological stages. (**A**) Florescence, (**B**) Fruiting, (**C**) Emergence, (**D**) Relaxation.

We compared the δ^13^C and δ^15^N values among the two *Bletilla* species and corresponding autotrophic reference plants at different phenological stages (Figure 6). The mean δ^13^C values varied significantly among the three groups across the four phenological stages. At the florescence and fruiting periods, the mean δ^13^C values of *B. striata* were highest, followed by *B. ochracea* and lastly the reference plants, with significant difference among each of the three groups. At the emergence and relaxation periods, the mean δ^13^C values of *B. ochracea* and *B. striata* were significantly higher than that of reference plants, but the two species did not significantly differ (Table 1). For the mean values of δ^15^N, significant differences among the three groups only existed in the florescence and fruiting periods. At florescence, *B. striata* and *B. ochracea* had significantly higher δ^15^N values than that of reference plants, while at fruiting, the two *Bletilla* species had significantly lower mean δ^15^N values than that of reference plants (Table 1).

The δ^13^C and δ^15^N values among different phenological stages were also compared for each plant group (Table 1). For the values of δ^13^C, significant differences among the four phenological stages only existed in *B. ochracea* and autotrophic reference plants. For *B. ochracea*, the emergence and relaxation periods had significantly higher mean δ^13^C values than that of fruiting period. For the values of δ^15^N, significant differences among the four phenological stages existed for all the three plant groups. The fruiting period had significantly lower δ^15^N values than other three periods for both *Bletilla* species.

### 2.5. Enrichment Factors and N Concentrations of Two Bletilla Species at Different Phenological Stages

**Figure 7:**
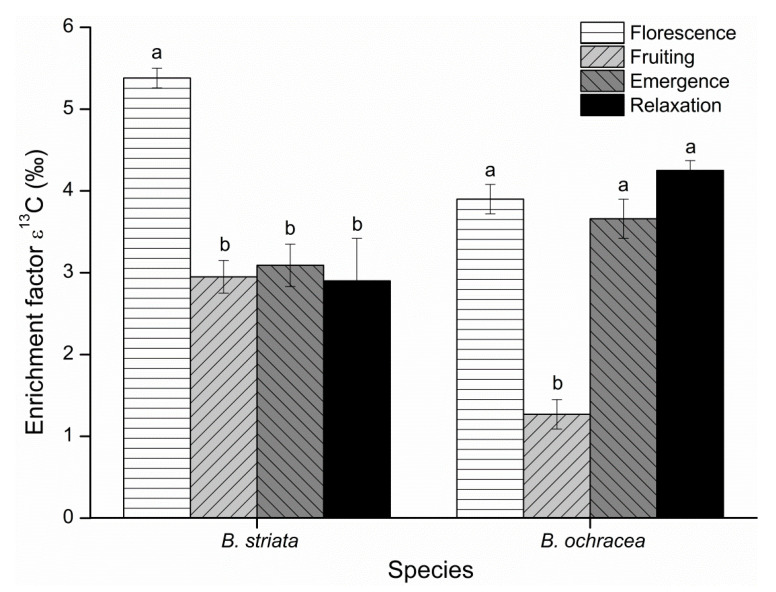
Enrichment factor (ε) for ^13^C of *Bletilla striata* and *B*. *ochracea* collected at different phenological stages. Mean ε values of the autotrophic reference species are equal to zero. Different lowercase letters for each species indicate statistically significant differences among phenological stages (*p* < 0.05).

**Figure 8:**
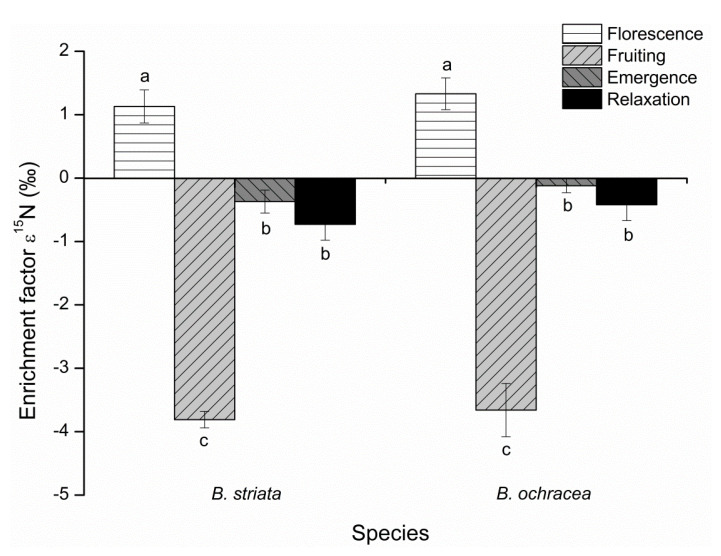
Enrichment factor (ε) for ^15^N of *Bletilla striata* and *B*. *ochracea* collected at different phenological stages. Mean ε values of the autotrophic reference species are equal to zero. Different lowercase letters for each species indicate statistically significant differences among phenological stages (*p* < 0.05).

Relative enrichment factors (ε) for ^13^C and ^15^N were calculated for the two *Bletilla* species at four phenological stages. For *B. striata*, the ε^13^C value at florescence was significantly higher than those at emergence, fruiting and relaxation, and there were no significant differences among the latter three. For *B. ochracea*, the situation is rather different, with the ε^13^C values during relaxation, florescence and emergence periods being significantly higher than that during fruiting (Figure 7). However, the ε^15^N values at the different phenological stages for the two *Bletilla* species had the same temporal trend, with the values at florescence significantly higher than those at emergence and relaxation, and the values at emergence and relaxation being significantly higher than that at fruiting (Figure 8). The ε^13^C values confirmed a significant ^13^C enrichment for the two *Bletilla* species at the four phenological stages. The ε^15^N values confirmed significant ^15^N enrichment during florescence, with significant ^15^N depletion during fruiting for the two *Bletilla* species.

We also compared the ε^13^C and ε^15^N differences of the two *Bletilla* species at each phenological stage. At florescence and fruiting, *B. striata* had higher ε^13^C values than *B. ochracea*, while *B. striata* had lower ε^13^C values than *B. ochracea* at the emergence and relaxation stages. Meanwhile, based on ε^15^N values, *B. ochracea* had higher enrichment during florescence and less depletion at fruiting, emergence and relaxation stages compared with *B. striata*, though the differences were rather small.

**Figure 9:**
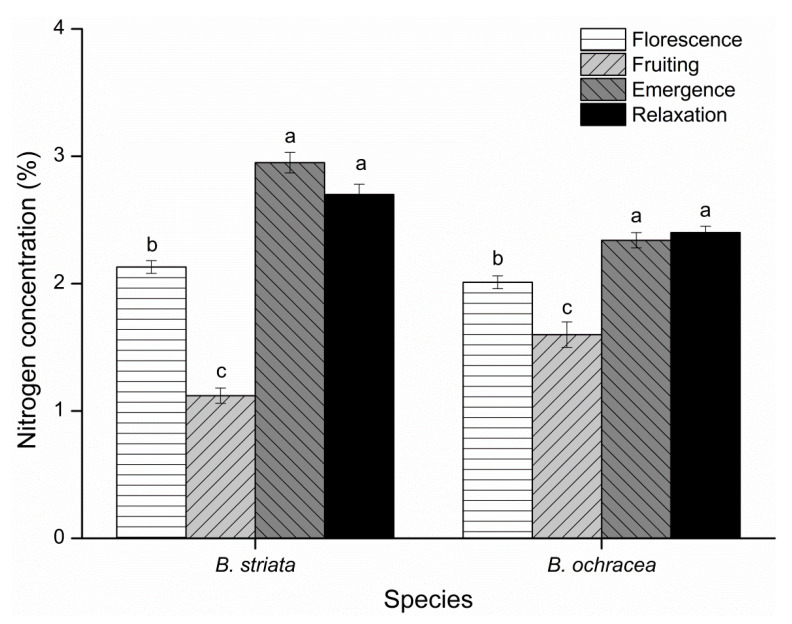
Temporal variation in leaf nitrogen concentration for *Bletilla striata* and *B*. *ochracea*. Different lowercase letters for each species indicate statistically significant differences among phenological stages (*p* < 0.05).

Nitrogen concentrations were compared among different phenological stages for *B. striata* and *B. ochracea*. For the two species, the nitrogen concentrations at the emergence and relaxation periods were significantly higher than those during florescence, while the values during florescence were significantly higher than those during fruiting. Meanwhile, there were no significant differences between the values during the emergence and relaxation periods (Figure 9).

## 3. Discussion

### 3.1. Temporal Variation in Root-Associated Fungal Diversity for Bletilla Species 

In this study, significant temporal variation was observed in the diversity of root endophytic fungi associated with the two *Bletilla* species. Compared with the fruiting and dormancy periods, the florescence period of *B. striata* and the relaxation period of *B. ochracea* had significantly higher Shannon index values. This finding may be associated with dynamic changes in plant physiological process and interactions between root endophytic fungi and orchids because metabolic rate of plant and nutritional requirement vary among different physiological stages. Additionally, pelotons formed by orchid endophytic fungi are short-lived structures that undergo rapid turnover within orchid cells, allowing new colonization events [37]. Thus, the diversity of symbiotic fungi can also change quite rapidly [25]. Accordingly, temporal variation in fungal diversity across seasons has been found in many cases [38,39]. For example, Koide et al. [38] found variation in fungal diversity of ectomycorrhizae roots, and Jumpponen [39] found temporal changes in arbuscular mycorrhizal roots. In our study, *Bletilla* plants become dormant with extremely low metabolic rate during dormancy period, which may lead to the lowest root endophytic fungal diversity in this period. The fruiting period had lower root endophytic fungal diversity, which may be due to the weaker physiological activity, higher temperature or lower soil water content during summer. The senescence of plant accompanying weaker physiological activity during fruiting periods, which do not need too much nutrition supply, may reduce the dependence of plants on fungi [40]. Meanwhile, high temperature and drought would reduce microbial population size and thus restrain community diversity and complexity [41]. Tedersoo et al. [42] found that although host plant families had strong effects on the phylogenetic community composition of fungi, temperature and precipitation mostly affected ectomycorrhizal fungal richness. Han et al. [43] found that *Paphiopedilum spicerianum* was associated with obviously more mycorrhizal fungi OTUs with higher Shannon values during wet seasons (274 and 1.88, respectively) relative to dry seasons (143 and 1.54, respectively). 

Significant temporal variation in root-associated fungal endophyte community structure across seasons was also found in this study. During florescence, *B. striata* and *B. ochracea* were dominated by Ascomycota, while during fruiting, dormancy and relaxation Basidiomycota were dominant. However, during emergence, *B. striata* fungal associations were dominated by Ascomycota, and *B. ochracea* fungal associations were dominated by Basidiomycota. Meanwhile, the abundance of dominant species at various phenological stages varied among *Bletilla* species. The reason root endophytic fungi community composition and its abundance varied among phenological stages requires further analysis. There are three possible reasons. First, some root endophytic fungi require metabolites produced by orchid plants at some particular period for its growth [19]. Second, mycorrhizal tissue structure varies among physiological periods, underlying different mycorrhizal fungi infection rates and fungi types. Third, the ecological factors extrinsic to host plant roots, such as precipitation, light irradiation, soil temperature and humidity, can vary among physiological periods, greatly influencing the root endophytic community composition. The reasons for different responses of fungi community composition and abundances to phenological changes by two species are difficult to identify. One most likely explanation may be genetic differences in the plant species. Tedersoo et al. [42] found that host plant family had the strongest effect on the phylogenetic community composition of fungi. 

At the family level, the florescence stage for both *Bletilla* species had the highest fungal diversity, likely owing to orchid plants requiring considerable nutrients to bloom, exceeding the nutrients supplied by photosynthesis; accordingly, they may need root endophytic fungi to supply nutrients to enable blooming [44]. Data of ε^13^C and ε^15^N in this study substantiate this finding. Bellino et al. [45] found that phototrophic and mycotrophic nutrition alternate change with the seasons and nutritional requirements of plants. Another possible explanation is that the outer velamen tissue of orchids is destroyed to some extent during the fast-growing stages through friction with soil, which results in massive fungal invasion during later physiological stages (e.g., florescence). Thus, root cells are heavily colonized and have high activity of invading hyphae, which leads to the highest fungal infection rate occurring at the florescence stage. Rasmussen et al. [46] found that mycorrhizal infection reached its maximum intensity 2–6 months after mycotrophic root development.

In this study, the composition and abundance of total species, dominant species and endemic species varied between the two *Bletilla* species at each phenological stage and also varied among phenological stages for both species. This may reflect distinct mycorrhizal preferences for specific host species, or alternatively, this pattern could result from hosts undergoing natural selection for different fungal endophytes enabling better adaption to their environments [47,48].

### 3.2. Isotopic Abundance Trends over the Growth Season

Our results demonstrate that variation in ^13^C and ^15^N enrichment can be highly dynamic throughout the whole growth season for *Bletilla* spp. For *B. striata*, the florescence period had significantly higher ε^13^C values, while the ε^13^C value during fruiting in *B. ochracea* was significantly lower than that during the other three periods. For the two *Bletilla* species, the ε^15^N values during florescence were significantly higher than those during emergence and relaxation periods, and the values at the emergence and relaxation periods were significantly higher than that of the fruiting period. This indicates that *Bletilla* plants substantially change their isotopic content across phenological stages, which is inconsistent with the results of Ercole et al. [25], who reported only slight changes in ^13^C and ^15^N natural abundances in the evergreen species *Anacamptis morio* throughout all its growth stages. 

In this study, the two *Bletilla* species were relatively enriched in ^13^C across all phenological stages (except dormancy), indicating that they obtained carbon from their root endophytic fungi throughout the whole year. Because fungal tissues have higher ^13^C stable isotope abundance compared with neighbouring autotrophic plants [26,27], according to the isotopic differences between plant- and fungus-derived C, we can trace the source of carbon by ^13^C enrichment across different phenological stages. Additionally, *B. striata* had higher ε^13^C values at florescence and fruiting and lower ε^13^C values at emergence and relaxation compared with *B. ochracea*, which indicates that the carbon obtained from root endophytic fungi varied among *Bletilla* species and phenological stages. This variation in ^13^C enrichment may be owing to variation in climate, genetic effects, soil nutrients and/or mycorrhizal associations [17,49,50]. However, in this study, soil total carbon, nitrogen, phosphorus content and pH did not differ substantially among *B. striata* and *B. ochracea* sites (Table S2). The observed interspecific variation in ^13^C enrichment at same time might owing to genetic variation within *Bletilla* spp. Meanwhile, the variation in ^13^C enrichment among phenological periods might be owing to variations in mycorrhizal tissue structure and ecological factors such as soil temperature, soil humidity and light radiation, which greatly influenced the ease with which fungi infect roots as well as root endophytic fungi types, thus affecting root endophytic fungal community composition. Additionally, ^13^C enrichment was positively correlated with the abundances of Ascomycota at the phylum level across all growth stages. Selosse et al. [51] found that Ascomycota species were often found in orchid roots and could form typical orchid mycorrhizae.

Obvious enrichment of ^15^N during the florescence period and significantly strong ^15^N depletion at the fruiting period were found for the two *Bletilla* species, indicating that *Bletilla* species obtained N from their mycorrhizal fungi during florescence and that a significant transfer of nitrogen from orchids to their mycorrhizal associates occurred during the fruiting period. Researchers found that a transfer of nutrients from the orchids to their mycorrhizal associates was consistent with such ^13^C and ^15^N depletions [52,53]. Meanwhile, compared with *B. striata*, *B. ochracea* had relatively higher enrichment during florescence and lower depletion of ^15^N at other phenological stages, indicating that *B. ochracea* obtained more nitrogen from their associated mycorrhizal fungi during the florescence period and transferred less N to mycorrhizal fungi during other phenological stages compared with *B. striate*.

For both *B. striata* and *B. ochracea*, leaf total nitrogen concentrations were significantly lower during the florescence and fruiting periods compared with the emergence and relaxation periods, which is most probably explained by nitrogen investments in flower and seed tissues. Liebel et al. [17] reported that compared with non-flowering/non-fruiting individuals, flowering/fruiting *Goodyera repens* individuals had lower leaf total nitrogen and chlorophyll concentrations, which is most probably explained by the plants using leaf nitrogen to form flowers and seeds. Andersson [54] showed that floral investment by *Nigella sativa* could cause reduced allocation to other plant functions. Based on the obvious enrichment of ^15^N during florescence and significantly strong ^15^N depletion during fruiting, we can infer that *Bletilla* species might compensate for low leaf total nitrogen concentrations by using ^15^N enriched fungal sources during flowering but that there was no such compensation during fruiting. One explanation is a relative paucity of root endophytic fungal species and lower activity owing to drought stress during the fruiting period. Another likely reason is that photosynthesis can mostly support fruiting costs and thus maintain the same seed production by increasing leaf, stem and fruit photosynthesis without increases in carbon fluxes from fungus to orchid [16,17]. 

## 4. Materials and Methods 

### 4.1. Plant Species

*Bletilla* Rchb. f. is a genus of terrestrial orchids distributed across northern Burma, China and Japan, and it belongs to the tribe Epidendreae in the subfamily Orchidoideae within Orchidaceae [55]. *B. striata* and *B. ochracea* are two relatively widespread *Bletilla* species in China that are widely used for garden landscaping as well as traditional Chinese medicine [56]. Mature *Bletilla* plants enter dormancy in the winter and begin to bolt in the early spring under natural conditions. Adult plants form mycorrhizal associations, mainly with Ascomycota and Basidiomycota and are characterized by the phenological stages described in Table 2.

### 4.2. Study Site 

Samples were collected from the *Bletilla* germplasm resource nursery located at Shanghai Chenshan Botanical Garden (31°04′ N and 121°11′ E). The area has a subtropical monsoon climate with a cumulative mean annual precipitation of 1213 mm and a mean annual temperature of 15.6 °C. The soil matrix (within the top 0–5 cm) was composed of mountain clay, river sand and vermiculite, with a ratio of 1.5:1:1 and a pH of 6.5–7.5. The *Bletilla* plants examined had been cultivated for three years and were already fully established at the site.

### 4.3. Sampling

Sampling was conducted from May 2018 to April 2019 in the germplasm resource nursery of Shanghai Chenshan Botanical garden. Root samples were collected at five time points, while leaf samples were collected at four time points (outside of the dormancy period) spanning the different phenological stages (Table 2). Three 1 × 1 m plots were randomly established in the *B. striata* and *B. ochracea* sites. In each plot, two roots per plant from five orchid plants were collected. Collected roots from each plot were pooled and kept cold during transport to the laboratory for further analysis. For each species, we sampled fresh top leaves from several *Bletilla* individuals at four time points (five individuals at florescence and fruiting periods and three individuals at emergence and relaxation periods). Meanwhile, leaves of three to five autotrophic reference plants under the same microclimate were also sampled at each phenological stage. The autotrophic reference species were chosen based on the criteria described by Gebauer and Meyer [23]. For detailed information on reference species, please see Table 1. In total, 30 root samples and 32 leaf samples from two *Bletilla* species and 59 leaf samples from autotrophic reference species were collected in this study.

### 4.4. Identification of Root Endophytic Fungi

Roots were rinsed with tap water, sonicated to remove any adhering soil and dirt and sterilized as follows: Roots were rinsed with sterile water for 30 s and then 70% ethyl alcohol for 2 min, soaked in 2.5% sodium hypochlorite for 5 min, transferred to 70% ethyl alcohol for 30 s and finally washed with sterile water three times. Roots were then cut into small pieces with sterile scissors, and ten to twelve sections per sample were selected for genomic DNA extraction and purification using the FastDNA Spin Kit for Soil (MP Biomedicals, Irvine, CA, USA) according to the manufacturer’s protocol. The nuclear ribosomal internal transcribed spacer (ITS) region was amplified with the fungal-specific primers ITS1F and ITS4 [17]. The PCR amplification was performed as follows: initial denaturation at 95 ℃ for 3 min, followed by 35 cycles of denaturing at 95 ℃ for 30 s, annealing at 55 ℃ for 30 s and extension at 72 ℃for 45 s, single extension at 72 ℃ for 10 min, and end at 10 ℃. The PCR reactions were performed in triplicate 20 μL mixture containing 2 μL of 10 × Pyrobest Buffer, 2 μL of 2.5 mM deoxyribonucleotide triphosphates (dNTPs), 0.8 μL of each primer (5 μM), 0.2 μL of Pyrobest DNA Polymerase (TaKaRa), 10 ng of template DNA, and finally ddH_2_O up to 20 μL. The PCR product was extracted from 2% agarose gel and purified using the AxyPrep DNA Gel Extraction Kit (Axygen Biosciences, Union City, CA, USA) according to manufacturer’s instructions and quantified using Quantus™ Fluorometer (Promega, Madison, WI, USA).

All positive PCR products were purified with the AxyPrep DNA Gel Extraction Kit (Axygen Biosciences, Union City, CA, USA) and sequenced bidirectionally using an Illumina MiSeq PE300 platform (Illumina, San Diego, CA, USA) according to the standard protocols from Majorbio Bio-Pharm Technology Co. Ltd. (Shanghai, China). The generated paired-end reads were merged once, but because the reads were longer than 300 bp, the paired-end reads could not be merged without overlap. Thus, we used single-end long reads for further analysis. The raw reads were deposited into the NCBI Sequence Read Archive (SRA) database (Accession Number: SRP225764). Raw fastq files were demultiplexed and quality-filtered with Trimmomatic according to the following criteria: 300 bp reads that were truncated at any site receiving an average quality score of <20 over a 50 bp sliding window, and the truncated reads shorter than 50 bp were discarded; reads containing ambiguous characters were also discarded. Operational taxonomic units (OTUs) were clustered with a 97% similarity cut-off using UPARSE (version 7.1, http://drive5.com/uparse/), and chimeric sequences were removed using UCHIME. The taxonomy of each ITS rRNA gene sequence was analysed with the RDP Classifier algorithm (http://rdp.cme.msu.edu/) against the Unite 8.0 ITS rRNA database using a confidence threshold of 70%. Fungal OTUs were classified into different putative trophic strategies following the classification of the FunGuild v1.0 (http://www.stbates.org/guilds/app.php).

### 4.5. Analysis of Stable Isotope Abundance and N Concentration

Leaf samples were washed with deionized water, oven-dried at 105 °C, ground into a fine power and stored in a desiccator fitted with silica gel until subsequent analysis. Relative C and N isotope abundances and N content were measured using an elemental analyser (vario PYRO cube; Elementar Analysensysteme GmbH, Langenselbold, Germany) coupled with a continuous flow isotope ratio mass spectrometer (IsoPrime100, Elementar UK Ltd., Stockport, UK), as described by Bidartondo et al. [57]. Relative isotope abundances are denoted as δ values, which were calculated according to the equation:

(1) δ13C or δ15N=RsaRst−1×1000‰,

where *R*_sa_ and *R*_st_ are the ratios of heavy isotopes to light isotopes in the samples and the respective standards. Standard gases were calibrated with USGS40 and USGS41a for carbon and nitrogen isotopes, provided by the United States Geological Survey (USGS). Reproducibility and accuracy of the isotope abundance measurements were routinely controlled by measures of laboratory standard acetanilide. The calculation of N concentrations in the samples followed the protocol by Gebauer and Schulze [58]. For relative C and N isotope natural abundance analyses, acetanilide was routinely analysed with variable sample weights once every 12 samples.

Enrichment factors (ε_s_) for all samples were calculated according to the equation:

(2) $$\varepsilon_{s}=\delta_{s}-\delta_{\mathrm{ref}}$$

where δ_S_ is the relative isotope abundance of a *Bletilla* sample, and δ_ref_ is the mean isotope abundance of all autotrophic reference plants.

### 4.6. Statistical Analyses 

Isotopic data analyses were performed using SPSS 16.0 for Windows (SPSS Inc., Chicago, IL, USA). Before analysis, all variables were checked for normality by Shapiro–Wilks test and for homogeneity of variance by Levene’s test. Two-way ANOVA with Tukey HSD post hoc comparisons or Tamhane’s T2 test were used when data were normally distributed. We checked the differences in mean δ^13^C and δ^15^N values among the autotrophic reference plants, *B. striata* and *B. ochracea* as well as differences among different physiological stages for each species. Additionally, values of ε^13^C, ε^15^N and N concentration among the different physiological stages were also compared for each *Bletilla* species. Significant differences in three alpha diversity indexes (Sobs, Shannon and Simpson) and coverage among different physiological stages were also compared in this study. Significance was defined at the 95% confidence level throughout this article. Additionally, microbial species composition analysis was performed using the free online Majorbio I-Sanger Cloud Platform (www.i-sanger.com).

## 5. Conclusions

Identifying the trophic relationship of endangered *Bletilla* with its root endophytic partners is critical for understanding plant growth and ultimately restoring wild populations. We investigated temporal variation in root endophytic fungal diversity, as well as the natural abundance of carbon and nitrogen stable isotopes in two *Bletilla* species (*B. striata* and *B. ochracea*) at different phenological stages. Our results suggest that variations in ^13^C and ^15^N natural abundance and root endophytic fungi for *Bletilla* spp. can be highly dynamic across all phenological stages. Both *Bletilla* species obtained carbon from their mycorrhizal fungi during the whole year. *B. striata* obtained more carbon during the florescence and fruiting periods but obtained less carbon during the emergence and relaxation periods compared with *B. ochracea*. For both *Bletilla* species, the florescence stage had relatively more ^13^C and ^15^N enrichment compared with the other phenological stages. Due to the two-way selection of plant species and root endophytic fungi, the community structure and abundance of total species, dominant species and endemic species varied between the two *Bletilla* species and among phenological stages. Additionally, the varying tendency of root endophytic fungal diversity across the whole growth stage was consistent with ^13^C enrichments, which was also accord with the nutritional requirement of plant. Our results have important implications for the current understanding of fungus–host relationships and also provide practical information for *Bletilla* conservation efforts.

## Figures and Tables

**Table 1:** Comparison of δ^13^C and δ^15^N values (mean ± SE) among different species at each phenological stage and different phenological stages for each species.

	δ^13^C (‰)				δ^15^N (‰)			
	Florescence	Fruiting	Emergence	Relaxation	Florescence	Fruiting	Emergence	Relaxation
*B. striata*	−26.02 ± 0.12 ^aA^	−27.22 ± 0.2 ^aA^	−27.1 ± 0.26 ^aA^	−27.29 ± 0.52 ^aA^	1.46 ± 0.26 ^aA^	−0.63 ± 0.13 ^bB^	1.51 ± 0.18 ^aA^	1.15 ± 0.25 ^aA^
*B. ochracea*	−27.5 ± 0.18 ^bAB^	−28.9 ± 0.18 ^bB^	−26.53 ± 0.24 ^aA^	−26.93 ± 0.12 ^aA^	1.66 ± 0.25 ^aA^	−0.48 ± 0.42 ^bB^	1.76 ± 0.13 ^aA^	1.83 ± 0.25 ^aA^
ref	−31.4 ± 0.22 ^cC^	−30.17 ± 0.24 ^cA^	−30.19 ± 0.20 ^bAB^	−31.18 ± 0.27 ^bBC^	0.33 ± 0.25 ^bC^	3.18 ± 0.32 ^aA^	1.88 ± 0.52 ^aB^	2.25 ± 0.47 ^aAB^

Values followed by the same lowercase letters within a column did not have significantly different δ^13^C or δ^15^N values. Values followed by the same capital letters within a row did not have significantly different δ^13^C or δ^15^N values.

**Table 2:** Phenological stages and sample times of *Bletilla* and autotrophic reference plants.

Season	Phenological Stage	Species	Sampling Dates	Leaf Samples (*Bletilla* Individuals)	Reference Plant Species
Late spring	Flourishing with eighty percent flowers bloom.	*B. striata*	04-May-2018	5	*Erigeron annuus* (L.) Pers., *Bischofia javanica* Bl., *Morus alba* L., *Broussonetia papyrifera* (Linn.) LHer. ex Vent., *Conyza canadensis* (L.) Cronq.
		*B. ochracea*	14-May-2018	5	
Summer	Fruiting with capsules mature but closed.	*B. striata*	27-Aug-2018	5	*Cirsium setosum* (Willd.) MB., *Celtis sinensis* Pers., *Metaplexis japonica* (Thunb.) Makino, *Morus alba* L.
		*B. ochracea*	27-Aug-2018	5	
Winter	Plant dormancy. Leaves and floral stem have been dried out.	*B. striata*	18-Dec-2018	0	NA
		*B. ochracea*	18-Dec-2018	0	
Early spring	Buds emergence with leaves stacked together, and the tuber produces some roots.	*B. striata*	13-Mar-2019	3	*Cirsium setosum*, *Sonchus oleraceus* L., *Conyza canadensis*
		*B. ochracea*	25-Mar-2019	3	
Middle spring	Shoots developed with leaves expanded, and floral stems are produced.	*B. striata*	02-Apr-2019	3	*Sonchus oleraceus*, *Conyza canadensis*, *Solanum nigrum* L.
		*B. ochracea*	17-Apr-2019	3	

## Supplementary Materials

The following are available online at https://www.mdpi.com/2223-7747/10/1/18/s1, Figure S1: Microbial community pieplots of fungi species shared among all five phenological stages in association with *Bletilla striata* (A) and *B. ochracea* (B) on genus level. Table S1: Temporal variation in mycorrhizal diversity associated with *Bletilla striata* and *B. ochracea.* Table S2: Soil nutrients and pH for *Bletilla striata* and *B. ochracea sites* (mean ± SE values).

## References

[1] Chase M.W., Cameron K.M., Freudenstein J.V., Pridgeon A.M., Salazar G., van den Berg C., Schuiteman A. (2015). An updated classification of Orchidaceae. Bot. J. Linn. Soc..

[2] Christenhusz M.J.M., Byng J.W. (2016). The number of known plants species in the world and its annual increase. Phytotaxa.

[3] McCormick M.K., Whigham D.F., O’Neill J. (2004). Mycorrhizal diversity in photosynthetic terrestrial orchids. New Phytol..

[4] Roberts D., Dixon K. (2008). Orchids. Curr. Biol..

[5] Zettler L.W., Sharma J., Rasmussen F., Dixon K., Cribb P., Kell S., Barrett R. (2004). Mycorrhizal diversity. Orchid Conservation.

[6] Jin W.T., Xiang X.G., Jin X.H. (2015). Generic delimitation of Orchidaceae from China: Current situation and perspective. Biodivers. Sci..

[7] Bailarote B.C., Lievens B., Jacquemyn H. (2012). Does mycorrhizal specificity affect orchid decline and rarity?. Am. J. Bot..

[8] Waterman R.J., Bidartondo M.I. (2008). Deception above, deception below: Linking pollination and mycorrhizal biology of orchids. J. Exp. Bot..

[9] Swarts N.D., Dixon K.W. (2009). Terrestrial orchid conservation in the age of extinction. Ann. Bot..

[10] Herrera H., Valadares R., Oliveira G., Fuentes A., Almonacis L., Bashan Y., Arriagada C. (2018). Adaptation and tolerance mechanisms developed by mycorrhizal *Bipinnula fimbriata* plantlets (Orchidaceae) in a heavy metal-polluted ecosystem. Mycorrhiza.

[11] Smith S.E., Read D.J. (2008). Mycorrhizal Symbiosis.

[12] Schiebold J.M.I., Bidartondo M.I., Karasch P., Gravendeel B., Gebauer G. (2017). You are what you get from your fungi: Nitrogen stable isotope patterns in Epipactis species. Ann. Bot..

[13] Stockel M., Tesitelova T., Jersakova J., Bidartondo M.I., Gebauer G. (2014). Carbon and nitrogen gain during the growth of orchid seedlings in nature. New Phytol..

[14] Schiebold J.M.I., Bidartondo M.I., Lenhard F., Makiola A., Gebauer G. (2018). Exploiting mycorrhizas in broad daylight: Partial mycoheterotrophy is a common nutritional strategy in meadow orchids. J. Ecol..

[15] Liu S.S., Chen J., Li S.C., Zeng X., Meng Z.X., Guo S.X. (2015). Comparative transcriptome analysis of genes involved in GA-GID1-DELLA regulatory module in symbiotic and asymbiotic seed germination of *Anoectochilus roxburghii* (Wall.) Lindl. (Orchidaceae). Int. J. Mol. Sci..

[16] Gonneau C., Jersakova J., de Tredern E., Till-Bottraud I., Saarinen K., Sauve M., Roy M., Hajek T., Selosse M.A. (2014). Photosynthesis in perennial mixotrophic *Epipactis* spp. (Orchidaceae) contributes more to shoot and fruit biomass than to hypogeous survival. J. Ecol..

[17] Liebel H.T., Bidartondo M.I., Gebauer G. (2015). Are carbon and nitrogen exchange between fungi and the orchid *Goodyera repens* affected by irradiance?. Ann. Bot..

[18] Burke R.M., Cairney J.W.G. (1998). Carbohydrate oxidases in ericoid and ectomycorrhizal fungi: A possible source of Fenton radicals during the degradation of lignocelluloses. New Photol..

[19] Slezack S., Dumas-Gaudot E., Rosendahl S., Kjoller R. (1999). Endoproteolytic activities in pea roots inoculated with the arbuscular mycorrhizal fungus Glomus mosseae and/or Aphanomyces euteiches in relation to bioprotection. New Photol..

[20] McCormick M.K., Taylor D.L., Juhaszova K., Burnett J.R., Whigham D.F., Oneill J.P. (2012). Limitations on orchid recruitment: Not a simple picture. Mol. Ecol..

[21] Hynson N.A., Madsen T.P., Selosse M.A., Adam I.K.U., Ogura-Tsujita Y., Roy M., Gebauer G., Merckx V.S.F.T. (2013). The physiological ecology of mycoheterotrophy. Mycoheterotrophy. The Biology of Plants Living on Fungi.

[22] Van der Heijden M.G.A., Bardgett R.D., van Straalen N.M. (2008). The unseen majority: Soil microbes as drivers of plant diversity and productivity in terrestrial ecosystems. Ecol. Lett..

[23] Gebauer G., Meyer M. (2003). ^15^N and ^13^C natural abundance of autotrophic and mycoheterotrophic orchids provides insight into nitrogen and carbon gain from fungal association. New Phytol..

[24] Dawson T.E., Mambelli S., Plamboeck A.H., Templer P.H., Tu K.P. (2002). Stable isotopes in plant ecology. Annu. Rev. Ecol. Syst..

[25] Ercole E., Adamo M., Rodda M., Gebauer G., Girlanda M., Perotto S. (2015). Temporal variation in mycorrhizal diversity and carbon and nitrogen stable isotope abundance in the wintergreen meadow orchid *Anacamptis morio*. New Phytol..

[26] Gebauer G., Dietrich P. (1993). Nitrogen isotope ratios in different compartments of a mixed stand of spruce, larch and beech trees and of understory vegetation including fungi. Isot. Isot. Environ. Health Stud..

[27] Gleixner G., Danier H.J., Werner R.A., Schmidt H.L. (1993). Correlations between the 13C content of primary and secondary plant products in different cell compartments and that in decomposing basidiomycetes. Plant Physiol..

[28] Taylor D.L., Bruns T.D., Szaro T.M., Hodges S.A. (2003). Divergence in mycorrhizal specialization within *Hexalectris spicata* (Orchidaceae), a nonphotosynthetic desert orchid. Am. J. Bot..

[29] Roy M., Watthana S., Richard F., Vessabutr S., Selosse M.A. (2009). Mycoheterotrophic orchids from Thailand tropical dipterocarpacean forests associate with a broad diversity of ectomycorrhizal fungi. BMC Biol..

[30] Yagame T., Orihara T., Selosse M., Yamato M., Iwase K. (2012). Mixotrophy of *Platanthera minor*, an orchid associated with ectomycorrhiza-forming Ceratobasidiaceae fungi. New Phytol..

[31] Martos F., Dulormne M., Pailler T., Bonfante P., Faccio A., Fournel J., Dubois M.P., Selosse M.A. (2009). Independent recruitment of saprotrophic fungi as mycorrhizal partners by tropical achlorophyllous orchids. New Phytol..

[32] Ogura-Tsujita Y., Gebauer G., Hashimoto T., Umata H., Yukawa T. (2009). Evidence for novel and specialised mycorrhizal parasitism: The orchid *Gastrodia confusa* gains carbon from saprotrophic Mycena. Proc. R. Soc. Lond. B Biol. Sci..

[33] Girlanda M., Segreto R., Cafasso D., Liebel H.T., Rodda M., Ercole E., Salvatore C., Gebauer G., Perotto S. (2011). Photosynthetic Mediterranean meadow orchids feature partial mycoheterotrophy and specific mycorrhizal associations. Am. J. Bot..

[34] Liebel H.T., Bidartondo M.I., Preiss K., Segreto R., Stockel M., Rodda M., Gebauer G. (2010). C and N stable isotope signatures reveal constraints to nutritional modes in orchids from the Mediterranean and macaronesia. Am. J. Bot..

[35] Phillips R.D., Barrett M.D., Dixon K.W., Hopper S.D. (2011). Do mycorrhizal symbioses cause rarity in orchids?. J. Ecol..

[36] Ogura-Tsujita Y., Gebauer G., Xu H., Fukasawa Y., Umata H., Tetsuka K., Kubota M., Schweiger J.M.I., Yamashita S., Maekawa N. (2018). The giant mycoheterotrophic orchid *Erythrorchis altissima* is associated mainly with a divergent set of wood-decaying fungi. Mol. Ecol..

[37] Peterson R.L., Uetake Y., Bonfante P., Faccio A. (1996). The interface between fungal hyphae and orchid protocorm cells. Can. J. Bot..

[38] Koide R.T., Durland L., Shumway D.L., Xu B., Sharda J.N. (2007). On temporal partitioning of a community of ectomycorrhizal fungi. New Phytol..

[39] Jumpponen A. (2011). Analysis of ribosomal RNA indicates seasonal fungal community dynamics in Andropogon gerardii roots. Mycorrhiza.

[40] Hou T.W., Jin H., Liu H.X., An D.J., Luo Y.B. (2010). The variations of mycorrhizal fungi diversity among different growing periods of the dominant orchids from two habitats in the Huanglong valley, Sichuan. Acta Ecol. Sin..

[41] Zhang X.M., Johnston E.R., Li L.H., Konstantinidis K.T., Han X.G. (2017). Experimental warming reveals positive feedbacks to climate change in the Eurasian Steppe. ISME J..

[42] Tedersoo L., Bahram M., Toots M., Diedhiou A.G., Henkel T.W., Kjoller R., Morris M.H., Nara K., Nouhra E., Peay K.G. (2012). Towards global patterns in the diversity and community structure of ectomycorrhizal fungi. Mol. Ecol..

[43] Han J.Y., Xiao H.F., Gao J.Y. (2016). Seasonal dynamics of mycorrhizal fungi in *Paphiopedilum spicerianum* (Rchb. f) Pfitzer-A critically endangered orchid from China. Glob. Ecol. Conserv..

[44] Lendenmann M., Thonar C., Barnard R.L., Salmon Y., Werner R.A., Frossard E., Jansa J. (2011). Symbiont identity matters: Carbon and phosphorus fluxes between *Medicago truncatula* and different arbuscular mycorrhizal fungi. Mycorrhiza.

[45] Bellino A., Alfani A., Selosse M.A., Guerrieri R., Borghetti M., Baldantoni D. (2014). Nutritional regulation in mixotrophic plants: New insights from *Limodorum abortivum*. Oecologia.

[46] Rasmussen H.M., Whigham D.F. (2002). Phenology of roots and mycorrhiza in orchid species differing in phototrophic strategy. New Phytol..

[47] Bruns T.D., Bidartondo M.I., Taylor D.L. (2002). Host specificity in ectomycorrhizal communities: What do the exceptions tell us?. Integr. Comp. Biol..

[48] Hynson N.A., Preiss K., Gebauer G. (2009). Is it better to give than receive? A stable isotope perspective to orchid–fungal carbon transport in the green orchid species *Goodyera repens* and *G. oblongifolia*. New Phytol..

[49] Mujica M.I., Saez N., Cisternas M., Manzano M., Armesto J.J., Perez F. (2016). Relationship between soil nutrients and mycorrhizal associations of two *Bipinnula* species (Orchidaceae) from central Chile. Ann. Bot..

[50] Sakamoto Y., Ogura-Tsujita Y., Ito K., Suetsugu K., Yokoyama J., Yamazaki J., Yukawa T., Maki M. (2016). The tiny-leaved orchid *Cephalanthera subaphylla* obtains most of its carbon via mycoheterotrophy. J. Plant Res..

[51] Selosse M.A., Faccio A., Scappaticci G., Bonfante P. (2004). Chlorophyllous and achlorophyllous specimens of *Epipactis microphylla* (Neottieae, Orchidaceae) are associated with ectomycorrhizal septomycetes, including truffles. Microb. Ecol..

[52] Cameron D.D., Johnson I., Read D.J., Leake J.R. (2008). Giving and receiving: Measuring the carbon cost of mycorrhizas in the green orchid, *Goodyera repens*. New Phytol..

[53] Hynson N.A., Bruns T.D. (2009). Evidence of a myco-heterotroph in the plant family Ericaceae that lacks mycorrhizal specificity. Proc. R. Soc. Lond. B.

[54] Andersson S. (2005). Floral costs in *Nigella sativa* (Ranunculaceae): Compensatory responses to perianth removal. Am. J. Bot..

[55] Flora of China Editorial Committee (1999). Flora of China.

[56] Qian C.D., Jiang F.S., Yu H.S., Fu Y.H., Cheng D.Q., Gan L.S., Ding Z.S. (2015). Antibacterial biphenanthrenes from the fibrous roots of *Bletilla striata*. J. Nat. Prod..

[57] Bidartondo M.I., Burghardt B., Gebauer G., Bruns T.D., Read D.J. (2004). Changing partners in the dark: Isotopic and molecular evidence of ectomycorrhizal liaisons between forest orchids and trees. Proc. R. Soc. B Biol. Sci..

[58] Gebauer G., Schulze E.D. (1991). Carbon and nitrogen isotope ratios in different compartments of a healthy and a declining *Picea abies* forest in the Fichtelgebirge, NE Bavaria. Oecologia.

