# Supplementary material for: Temporal Variation in Community Composition of Root Associated Endophytic Fungi and Carbon and Nitrogen Stable Isotope Abundance in Two Bletilla Species (Orchidaceae)
Source: Plants (Basel). 2020 Dec 24;10(1):18. doi: 10.3390/plants10010018 (PMC7824424; doi:10.3390/plants10010018)
Supplement: Supplementary file 1 [file plants-10-00018-s001.pdf]

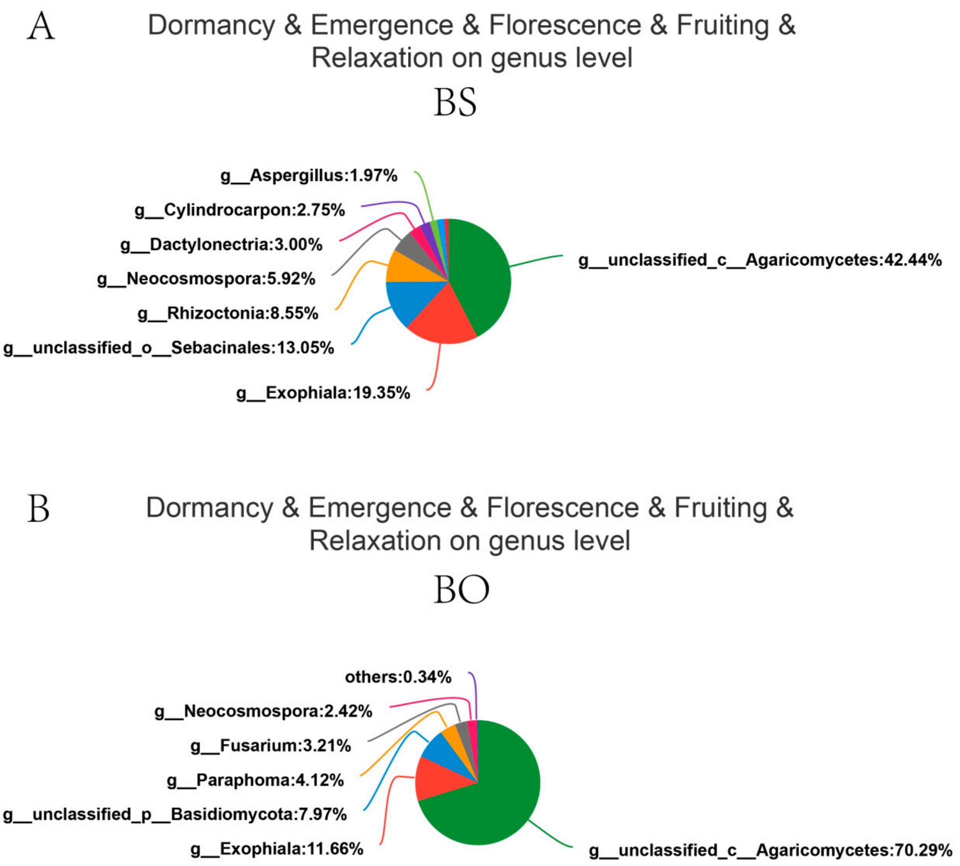

Figure S1. Microbial community pieplots of fungi species shared among all five phenological stages in association with *Bletilla striata* (**A**) and *B. ochracea* (**B**) on genus level.

Table S1. Temporal variation in mycorrhizal diversity associated with *Bletilla striata* and *B. ochracea*.

	Growth stage	Sobs index	Shannon index	Simpson's index	Coverage (%)
B. striata	Florescence	86 ± 17a	2.55 ± 0.33a	0.17 ± 0.05a	99.95a
	Fruiting	43 ± 10b	1.46 ± 0.33b	0.42 ± 0.14a	99.98a
	Dormancy	44 ± 3b	1.23 ± 0.42b	0.56 ± 0.16a	99.96a
	Emergence	55 ± 9ab	2.24 ± 0.13ab	0.2 ± 0.04a	99.97a
	Relaxation	31 ± 7b	1.58 ± 0.32ab	0.4 ± 0.14a	99.95a
B. ochracea	Florescence	66 ± 4ab	1.7 ± 0.19abc	0.34 ± 0.04ab	99.96a
	Fruiting	31 ± 8c	1.34 ± 0.13bc	0.4 ± 0.05ab	99.98a
	Dormancy	47 ± 6bc	1.23 ± 0.34c	0.5 ± 0.17a	99.96a
	Emergence	75 ± 4a	2.03 ± 0.15ab	0.27 ± 0.06ab	99.96a
	Relaxation	61 ± 8ab	2.34 ± 0.22a	0.19 ± 0.04c	99.97a

Values of each index across growth stages followed by different lowercase letters are significantly different ($p < 0.05$).

Table S2. Soil nutrients and pH for *Bletilla striata* and *B. ochracea* sites (mean ± SE values).

	TC (%)	TN (g/kg)	TP (g/kg)	pH
B. striata	9.97 ± 1.41	4.25 ± 0.58	1.14 ± 0.11	7.37
B. ochracea	10.86 ± 1.06	4.15 ± 0.36	1 ± 0.06	7.25

TC, total carbon; TN, total nitrogen; TP, total phosphorus
